# The potent and selective *α*4*β*2*/*α*6*-nicotinic acetylcholine receptor partial agonist 2-[5-[5-((*S*)Azetidin-2-ylmethoxy)-3-pyridinyl]-3-isoxazolyl]ethanol demonstrates antidepressive-like behavior in animal models and a favorable ADME-tox profile

**DOI:** 10.1002/prp2.26

**Published:** 2014-03-12

**Authors:** Li-Fang Yu, J Brek Eaton, Han-Kun Zhang, Emily Sabath, Taleen Hanania, Guan-Nan Li, Richard B van Breemen, Paul Whiteaker, Qiang Liu, Jie Wu, Yong-Chang Chang, Ronald J Lukas, Dani Brunner, Alan P Kozikowski

**Affiliations:** 1 Department of Medicinal Chemistry and Pharmacognosy, University of Illinois at Chicago833 South Wood Street, Chicago, Illinois, 60612; 2 Institute of Drug Design and Development, Shanghai Engineering Research Center of Molecular Therapeutics and New Drug Development, East China Normal University3663 North Zhongshan Road, Shanghai, 200062, China; 3 Division of Neurobiology, Barrow Neurological Institute350 West Thomas Road, Phoenix, Arizona, 85013; 4 Institute of Biomedical Sciences and School of Life Sciences, East China Normal University500 Dongchuan Road, Shanghai, 200241, China; 5 PsychoGenics, Inc.765 Old Saw Mill River Road, Tarrytown, New York, 10591; 6 Division of Neurology, Barrow Neurological Institute350 West Thomas Road, Phoenix, Arizona, 85013; 7 Department of Psychiatry, Columbia University, NYSPI1051 Riverside Drive, New York, New York, 10032

**Keywords:** Antidepressive-like behavior, nicotinic acetylcholine receptor, partial agonist, selectivity

## Abstract

Preclinical and clinical studies demonstrated that the inhibition of cholinergic supersensitivity through nicotinic antagonists and partial agonists can be used successfully to treat depressed patients, especially those who are poor responders to selective serotonin reuptake inhibitors (SSRIs). In our effort to develop novel antidepressant drugs, LF-3-88 was identified as a potent nicotinic acetylcholine receptor (nAChR) partial agonist with subnanomolar to nanomolar affinities for *β*2-containing nAChRs (*α*2*β*2, *α*3*β*2, *α*4*β*2, and *α*4*β*2*) and superior selectivity away from *α*3*β*4 − (*K*_i_ > 10^4^ nmol/L) and *α*7-nAChRs (*K*_i_ > 10^4^ nmol/L) as well as 51 other central nervous system (CNS)-related neurotransmitter receptors and transporters. Functional activities at different nAChR subtypes were characterized utilizing ^86^Rb^+^ ion efflux assays, two-electrode voltage-clamp (TEVC) recording in oocytes, and whole-cell current recording measurements. In mouse models, administration of LF-3-88 resulted in antidepressive-like behavioral signatures 15 min post injection in the SmartCube® test (5 and 10 mg/kg, i.p.; about 45-min session), decreased immobility in the forced swim test (1–3 mg/kg, i.p.; 1–10 mg/kg, p.o.; 30 min pretreatment, 6-min trial), and decreased latency to approach food in the novelty-suppressed feeding test after 29 days chronic administration once daily (5 mg/kg but not 10 mg/kg, p.o.; 15-min trial). In addition, LF-3-88 exhibited a favorable profile in pharmacokinetic/ADME-Tox (absorption, distribution, metabolism, excretion, and toxicity) assays. This compound was also shown to cause no mortality in wild-type Balb/CJ mice when tested at 300 mg/kg. These results further support the potential of potent and selective nicotinic partial agonists for use in the treatment of depression.

## Introduction

Depression is a common and frequently severe neurological condition, affecting ∼120 million people worldwide. Numerous therapeutic agents exist for the treatment of depression that target monoamine transporters selective for the neurotransmitters serotonin and norepinephrine (Berton and Nestler 2006). However, a considerable proportion of patients respond poorly to these drugs (Ruhe et al. 2006). The cholinergic hypothesis of depression holds that hyperactivity of the cholinergic system over the adrenergic system leads to depression (Janowsky et al. 1972). Preclinical and clinical studies have suggested the hypothesis that reduction in cholinergic supersensitivity with nicotinic antagonists and partial agonists has potential as a treatment for depression, especially for those patients who are poor responders to selective serotonin reuptake inhibitors (SSRIs) (George et al. 2008; Philip et al. 2009).

Two major types of cholinergic receptors are muscarinic ACh receptors (mAChRs) and nicotinic acetylcholine receptors (nAChRs), both of which are widely distributed in the central and peripheral nervous systems. The G protein-coupled mAChRs are believed to be involved in mood regulation and AChE-induced depressive behavior. Neuronal nAChRs belong to the ligand-gated ion channel superfamily of neurotransmitter receptors. Heteromeric *α*4*β*2*-nAChRs, the predominant form of nAChRs in the brain, are thought to have therapeutic benefits for a number of nervous system disorders in preclinical or clinical studies such as nicotine dependence, neuropathic pain, Alzheimer's disease, and Parkinson's disease (Hurst et al. 2013).

The nicotinic ligand having received the most attention as a potential antidepressant is mecamylamine. The first controlled study to show evidence that mecamylamine could ameliorate symptoms of mood disorders was a monotherapy study treating Tourette's disorder patients (Shytle et al. 2002b). Mecamylamine appeared to be particularly efficacious in the treatment of symptoms of major depression and showed significant improvements for subgroups with attention deficit hyperactivity disorder and hypomania, however, the subgroup sizes were very small, and the comorbidity with Tourette's disorder further precludes any conclusions being drawn about the efficacy of mecamylamine monotherapy in patients who do not have Tourette's disorder. Subsequent studies have investigated mecamylamine's efficacy as an augmentation therapy in combination with SSRIs or serotonin norepinephrine reuptake inhibitors (SNRIs) in treatment-resistant patients. Most notable are the clinical trials utilizing the positive enantiomer of mecamylamine, TC-5214. Targacept achieved favorable results in a Phase IIb clinical trial (Targacept press release, 2009), but collaborative efforts with AstraZeneca resulted in multiple Phase III trials that failed to meet their desired endpoints (AstraZeneca, 2012). While the Phase III failures do not disprove TC-2514's viability as a monotherapy, the compound lacks nAChR subtype selectivity and is not particularly potent. A recent estimate of the free mecamylamine concentration in brain indicates that it is likely on the edge pharmacological relevance, blocking ∼20% of *α*4*β*2*-nAChR function (Weber et al. 2013). This could explain inconsistencies between studies and illustrates the need for more potent nAChR compounds that are highly subtype-selective.

Targeting *α*4*β*2*-nAChRs (where the * indicates that nAChR subunits other than those indicated are or may be components of the receptor assembly) appears to be required for antidepressant effects of nicotinic ligands based on studies using nAChR subunit knockout animals and pharmacological approaches (Picciotto et al. 1998; Marubio et al. 2003; Caldarone et al. 2004; Rabenstein et al. 2006; Rollema et al. 2007). While *α*6 subunits are not widely distributed in the brain, they are, however, prevalent in midbrain dopaminergic regions in the mammalian central nervous system (CNS), indicating their potential association with depression (Klink et al. 2001; Shytle et al. 2002a; Quik et al. 2011; Wu and Lukas 2011). In the past, translation of nAChR research into viable pharmacotherapeutics has suffered in part due to the insufficient selectivity of ligands away from ganglionic *α*3*β*4*-nAChRs likely associated with unwanted side effects like emesis and nausea (Gotti et al. 2009). Cytisine derivatives have been shown to have relatively greater selectivity than cytisine itself for *β*2*-containing nAChRs over *α*3*β*4*nAChRs, and these agents have exhibited antidepressive-like behavior (Mineur et al. 2009). Sazetidine-A and its analogs have been found to exhibit excellent selectivity for *β*2-containing nAChRs over *α*3*β*4*- as well as *α*7-nAChRs. Replacing the acetylene bond with a substituted isoxazole ring lead to the identification of novel nicotinic ligands based on a pyridine ether scaffold that are more bioavailable in vivo (Caldarone et al. 2011; Yuan et al. 2013) while maintaining the key pharmacophoric elements of sazetidine-A. Guided by the hypothesis that selectivity for *β*2*-nAChRs by partial agonists is essential for antidepressive-like behavior in vivo, we report the characterization of a highly selective isoxazole-containing *β*2* partial agonist, namely LF-3-88, in a battery of pharmacological and behavioral assays, along with its preliminary pharmacokinetic/ADME-Tox (absorption, distribution, metabolism, excretion, and toxicity) profile.

## Materials and Methods

### Synthesis

**Figure 1:**
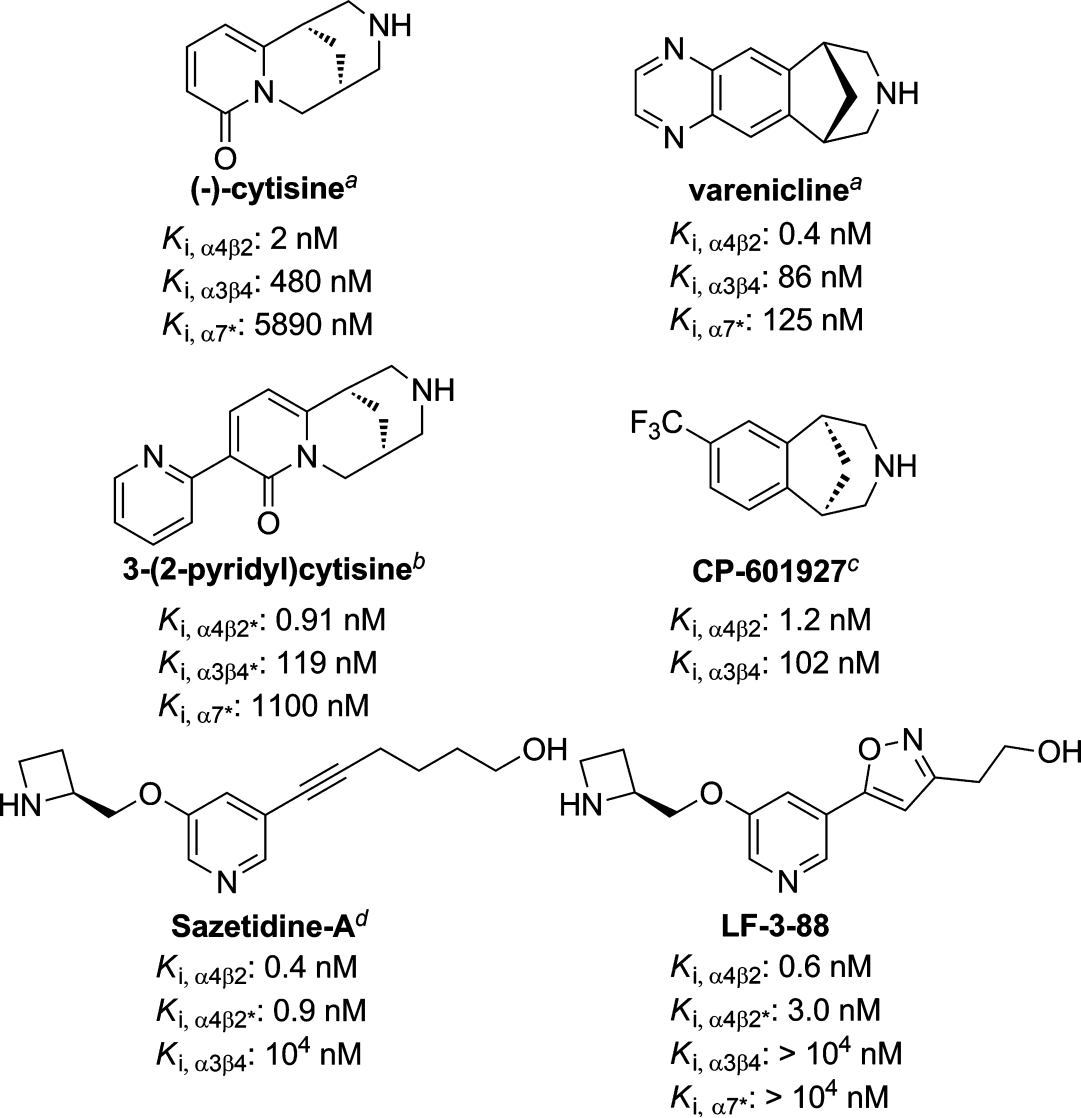
Structures of selected *α*4*β*2*-nAChR agonists. *K*_i_ values are taken from literature: ^*a*^Rollema et al. (2010), ^*b*^Mineur et al. (2009), ^*c*^Mineur et al. (2011), ^*d*^Xiao et al. (2006).

LF-3-88 is depicted in Figure 1 and was synthesized as described previously (Liu et al. 2011). In brief, the desired isoxazole was prepared via 1,3-dipolar cycloaddition of a nitrile oxide, derived from the corresponding nitro compound, to the appropriate alkyne. Subsequent deprotection of the precursor with trifluoroacetic acid (TFA) and purification by High-performance liquid chromatography (HPLC) yielded the desired final compound as its trifluoroacetate. Purity of the final compound (>98%) was established by analytical HPLC, which was carried out on an Agilent (Santa Clara, CA) 1100 HPLC system with a Synergi 4 *μ* Hydro-RP 80A column, with detection at 280 or 254 nm on a variable wavelength detector G1314A; flow rate = 1.4 mL/min; 18-min gradient of 0–100% methanol in water (both containing 0.05 vol% of TFA). The number of equivalents of TFA in this nonstoichiometric compound was determined by elemental analysis. All doses reported herein are expressed as the free base equivalents.

#### Reagents and chemicals

Compound LF-3-88 was synthesized as described above, and sertraline was purchased from Toronto Research Chemicals (Ontario, Canada). Compounds were administered by intraperitoneal (i.p.) injection in a volume of 10 mL/kg. Solvents including acetonitrile, methanol, and water were all Liquid chromatography–mass spectrometry (LC-MS) grade and purchased from Fisher Scientific (Fair Lawn, NJ). Formic acid was purchased from Sigma-Aldrich (St. Louis, MO).

### Radioligand-binding studies at CNS-related neurotransmitter receptors and transporters

A broad-range screening study was carried out for compound LF-3-88 at a 10 *μ*mol/L concentration to further determine its effects on 51 other CNS neurotransmitter receptors and transporters, including serotonin receptors, dopamine receptors, GABA receptors, biogenic amine transporters, adrenergic receptors, muscarinic receptors, opioid receptors, sigma receptors, and histamine receptors. For experimental details, please refer to the Psychoactive Drug Screening Program (PDSP) web site http://pdsp.med.unc.edu/.

## ^86^Rb ion efflux assays

^86^Rb ion efflux assays were used as previously described to characterize the functional effects of LF-3-88 (Lukas et al. 2002). Cell lines naturally or heterologously expressing specific, functional, human nAChR subtypes were used. The human clonal cell line TE671/RD naturally expresses human muscle-type *α*1*-nAChRs, containing *α*1, *β*1, *γ*, and *δ* subunits, with function detectable using ^86^Rb^+^ efflux assays. The human neuroblastoma cell line SH-SY5Y naturally expresses autonomic *α*3*β*4*-nAChRs, containing *α*3, *β*4, probably *α*5, and sometimes *β*2 subunits, and also displays function detectable using ^86^Rb^+^ efflux assays (Lukas et al. 1993). SH-SY5Y cells also express homopentameric *α*7-nAChRs, however, their function is not detected in the ^86^Rb^+^ efflux assay under the conditions used. SH-EP1 human epithelial cells stably transfected with human *α*4 and *β*2 subunits (SH-EP1-h*α*4*β*2 cells) have been established and characterized with both ion flux and radioligand-binding assays (Eaton et al. 2003). The recently established SH-EP1-h(*α*6/3)*β*2*β*3 cell line also was used (Breining et al. 2012). These cells heterologously express human nAChR chimeric (*α*6/3) subunits (wherein the extracellular, N-terminal, ligand-binding domain of the human nAChR *α*6 subunit replaces that domain in the nAChR *α*3 subunit) along with human *β*2 and *β*3 subunits. These cells express functional (*α*6/3)*β*2*β*3-nAChR that have pharmacological features of receptors containing wild-type *α*6 subunits, such as sensitivity to the *α*6*β*2*β*3-nAChR-selective functional antagonist, *α*-conotoxin MII (Kuryatov et al. 2000; McIntosh et al. 2004). TE671/RD, SH-SY5Y, and transfected SH-EP1 cell lines were maintained as low passage number (1–26 from frozen stocks) cultures to ensure stable expression of native or heterologously expressed nAChRs as previously described (Lukas et al. 2002). Cells were passaged once a week by splitting just-confluent cultures 1/300 (TE671/RD), 1/10 (SH-SY5Y), or 1/40 (transfected SH-EP1) in serum-supplemented medium to maintain log-phase growth.

Function of nAChR subtypes investigated was established as previously described (Lukas et al. 2002; Liu et al. 2011; Yu et al. 2012). One newer modification on these techniques involves low temperature incubation before assays to increase the surface expression of high sensitivity (HS) relative to low sensitivity (LS) *α*4*β*2 nAChRs and to enhance surface expression of (*α*6/3)*β*2*β*3-nAChRs. The ∼EC_90_ concentration of the full agonist carbamylcholine was 100 *μ*mol/L for SH-EP1-h(*α*6/3)*β*2*β*3 cells. Also, because SH-EP1-h*α*4*β*2 cells express a variable mixture of HS and LS h*α*4*β*2-nAChRs, and because sazetidine-A is a full agonist at HS h*α*4*β*2-nAChRs while having zero or nearly zero efficacy at LS h*α*4*β*2-nAChRs, as measured in the ^86^Rb^+^ ion flux assay, we determined efficacies of compounds studied at the two subtypes by comparing their effects normalized to responses to sazetidine-A and carbamylcholine, which is fully efficacious at both subtypes. In brief, this allows us to determine efficacies of test compounds at LS and HS *α*4*β*2 nAChRs, respectively, as the ordinate values of the linear regressions at abscissa values of 0% and 100% of sazetidine-A efficacy.

### Whole-cell current recording assays

Standard whole-cell current recording combined with instrumentation allowing rapid drug delivery and removal was utilized as described previously (Liu et al. 2008). In brief, SH-EP1-h*α*7 cells (Zhao et al. 2003) or SH-EP1-h*α*4*β*2 cells (Wu et al. 2004) were placed onto 35-mm culture dishes without poly(lysine) coating. Typically, after allowing the cells to grow for 4–6 days, culture dishes were placed on the stage of an inverted microscope (Axiovert 200; Zeiss, Germany), and a standard external solution (2 mL/min) was continuously superfused. Glass microelectrodes made with 3–5 MΩ resistance between pipette and extracellular solutions were used to obtain >1 GΩ seals on the cell surface just prior to conversion into standard whole-cell current recording mode by applying suction to the pipette. After allowing exchange between the pipette solution and the cytosol for 5 10 min, cells with attached pipettes were lifted gently from the bottom of the culture dish via micromanipulation of the pipette to improve kinetics of solution exchange around the cell. Access resistance (Ra) was measured and accepted if it was less than 20 MΩ. Pipette and whole-cell capacitance were both minimized, and series resistance was compensated routinely to 80%. Cells were voltage-clamped at −60 mV, and inward currents induced by indicated nicotinic agonists at the specified concentrations were measured (200B amplifier; Molecular Devices, Sunnyvale, CA). Current signals were typically filtered at 5 kHz, acquired at 10 kHz, displayed and digitized online (Digidata 1440A series A/D board; Molecular Devices). Data acquisition and analyses were conducted using pClamp9.2 (Molecular Devices), and results were plotted by using Origin 5.0 (OriginLab Corp., North Hampton, MA). All experiments were performed at room temperature (22 ± 1°C).

The external solution for SH-EP1 cell recording contained 120 mmol/L NaCl, 5 mmol/L KCl, 2 mmol/L MgCl_2_, 2 mmol/L CaCl_2_, 25 mmol/L d-glucose, and 10 mmol/L HEPES<< HEPES >>, pH 7.4 (Tris-base). For conventional whole-cell recording, the pipette solution contained 110 mmol/L Tris-phosphate dibasic, 28 mmol/L Tris-base, 11 mmol/L EGTA<< EGTA >>, 2 mmol/L MgCl_2_, 0.5 mmol/L CaCl_2_, and 4 mmol/L Na-ATP at pH 7.3. Drugs (standard compounds were obtained from Sigma Chemical, St. Louis, MO) were delivered to the recorded cell by a computer-controlled perfusion system (SF-77B Perfusion Fast Step, Warner Instruments Incorporated, A Harvard Bioscience Company, Holliston, MA), allowing the recorded cell can be completely surrounded by the applied drug within 20 msec. The interval between drug applications (2 min) was optimized specifically to ensure stability of nAChR responsiveness (without functional rundown).

### Two-electrode voltage-clamp

Xenopus oocytes were injected with cRNAs encoding human nicotinic receptor subunits (*α*4:*β*2 at 1:1 ratio or *α*7). Three to five days after injection, oocytes were individually placed in a custom made small volume chamber and continuously perfused with oocyte Ringer's solution (OR2; 92.5 mmol/L NaCl, 2.5 mmol/L KCl, 1 mmol/L CaCl_2_, 1 mmol/L MgCl_2_, and 5 mmol/L HEPES at pH 7.5). Drugs were delivered to the oocytes through a ValveLink8.2 computer-controlled perfusion system (Automate Scientific, Inc, Berkeley, CA). The chamber was grounded through an agar KCl bridge. The oocytes were voltage-clamped at −70 mV to measure agonist-induced currents using a GeneClamp 500B amplifier (Axon Instruments, Foster City, CA). The current signal was filtered at 20 Hz with the built-in four pole low-pass Bessel filter and digitized at 100 Hz.

#### Data analysis and statistics

To assess nAChR whole-cell current responses, both peak and steady-state currents were measured during the course of data acquisition and analyses. Results are shown as mean ± standard errors. Statistical analysis was applied using paired *t*-tests when assessing data obtained from a single cell, and Student's *t-*test (unpaired values) or one-way analysis of variance (ANOVA) with Duncan's multiple comparison were used to compare data obtained from different cells. Values of *P* less than 0.05 were considered significant. Curve fitting for agonist and antagonist concentration–response data were performed (Origin 5.0 software; OriginLab Corp.) using the logistic equation to provide fits for maximal and minimal responses, the EC_50_ or IC_50_ value, and Hill coefficients.

### Animals: SmartCube® test

Male C57 BL6 mice (Taconic Farms) were used in the SmartCube® assay (PsychoGenics Inc., Tarrytown, NY). Mice were received at 7 weeks old and group-housed in OptiMICE ventilated cages with four mice/cage. Mice were acclimated to the colony room for at least 1 week prior to the first injections. All animals were tested in SmartCube® at 8 10 weeks old.

### Animals: forced swim test

Male Balb/CJ mice (Jackson Laboratories, Bar Harbor, ME) were used in the forced swim test (FST). Upon receipt, mice were assigned unique identification numbers (tail marked) and were group-housed in OptiMICE ventilated cages. Mice were acclimated to the colony room for at least 1 week prior to drug administration. Mice were 8–10 weeks of age at time of FST.

### Animals: novelty-suppressed feeding test

Male C57 BL6/J (from Jackson Laboratories) mice were used for these studies. Upon receipt at 6 weeks of age, mice were single-housed in OptiMICE ventilated cages and acclimated to the colony room for 1 week prior to beginning of the dosing regimen at 7 weeks of age.

All mice were maintained on a 12/12 light/dark cycle with the light on at 8:00 am. The room temperature was maintained between 20°C and 23°C with a relative humidity between 30% and 70%. All animals were examined, handled, and weighed prior to initiation of the study to assure adequate health and suitability and to minimize nonspecific stress associated with manipulation. Food and water are provided ad libitum for the duration of the study. All animal experiments were conducted in accordance with the National Institutes of Health (NIH) Guide for the Care and Use of Laboratory Animals and the PsychoGenics Animal Care and Use Committee.

### General procedures for behavioral studies

#### SmartCube® test

To acquire initial in vivo evaluation of our nicotinic ligands for behavioral effects relevant to psychiatric diseases, we used SmartCube®, an automated system in which mouse behavior is captured by digital video using novel, proprietary hardware that presents multiple challenges in a single test session and is analyzed with computer algorithms. Digital videos of the subjects are processed with computer vision algorithms to extract more than 1400 dependent measures including frequency and duration of behavioral states such as grooming, rearing, etc., and many other features obtained during the test session. Using machine learning techniques chosen to best separate pharmacological effects of reference compounds, the behavioral signatures of the mutant mice are then assessed quantitatively.

#### Bioinformatics analysis

The most dominant of the features collected that define the drug class signature are identified and ranked using complex proprietary bioinformatics algorithms. The outcome of a SmartCube® run is a set of more than 1400 features (behavioral parameters) that can be used for various analyses. Many of these features are correlated (e.g., rearing counts and supported rearing counts). Therefore, we form statistically independent combinations of the original features (further referred to as decorrelated features) that discriminate between the groups more effectively. Next, we apply a proprietary feature ranking algorithm to score each feature discrimination power to weight each feature change by its relevance. The data obtained in this way are used to define drug signatures for known reference compounds and to establish therapeutic class signatures against which a test sample can be compared. The neuropharmacological effects of a test compound can be predicted by similarity to major classes of compounds, such as antipsychotics, anxiolytics, and antidepressants (Roberds et al. 2011).

#### Method

Mice are taken in their home cage to the SmartCube® suite of experimental rooms where they remain until they are placed in the apparatus for a single testing session. A standard SmartCube® protocol runs for a single session (∼45 min). After the session mice are group-housed again and brought back to the colony room. Any abnormal behavior is noted. Mice were injected with cytisine (0.5 and 1.0 mg/kg), varenicline (1.0 and 3.0 mg/kg), or LF-3-88 (5 and 10 mg/kg) 15 min before the test, during which multiple experimental challenges were presented over the course of the test session. At least 12 mice were used in each treatment group.

#### Forced swim test

Procedures have been previously described (Porsolt et al. 1977). Naïve mice were individually placed into clear glass cylinders (15 cm tall × 10 cm wide, 1 L beakers) containing 23 ± 1°C water 12 cm deep (∼800 mL). Mice were administered vehicle, the SSRI sertraline (10 or 20 mg/kg) as a positive control, or LF-3-88 (0.1, 0.3, 1, and 3 mg/kg, i.p.; or 1, 3, and 5 mg/kg, p.o.). Thirty minutes following the administration, mice were placed in the water, and the time the animal spent immobile was recorded over a 6-min trial. Immobility was defined as the postural position of floating in the water. Only one session is required for the forced swim test in mice.

#### Novelty suppressed feeding (NSF) test

Mice were tested in NSF on day 29 of treatment. Eighteen hours prior to behavioral testing, all food was removed from the home cage. The NSF test was carried out during a 15-min period. The test chamber consists of a rectangular (14″ × 20″ × 6″) plastic box. The chamber floor is covered with bedding (∼0.5″). At the time of testing, animals were placed in the left front corner of the chamber and two food pellets (regular chow) were placed on a laminated white paper circle (13 cm in diameter) positioned in the center of the test chamber. The latency to eat is defined by the animal holding the food with one or both paws and taking a bite of the pellet and staying in the center eating for at least 5 sec. The mice were videotaped and tapes were scored by two trained observers blind to the treatments. On test day (day 29), mice were pretreated with water or LF-3-88 30 min prior to testing, while imipramine was administered 60 min prior to testing. Upon completion of NSF, about 50 60 min after the pretreatment, mice were decapitated and all animals from all treatment groups except from the imipramine group were processed for collection of trunk blood and whole-brain tissue. Trunk blood was collected in microcentrifuge tubes (1.5 mL) containing K_2_EDTA and kept on ice for short-term storage. Within 15 min, the tubes were centrifuged immediately for 10 min at 10,000 12,000 rpm in a refrigerated centrifuge. Plasma was extracted and samples stored in the −80°C freezer before testing. For brain tissues, whole brains were deposited in microcentrifuge tubes (1.5 mL) on dry ice. Samples are stored at −80°C until analysis.

#### Quantitative analysis

Concentrations of LF-3-88 in the brain and plasma were determined using the previously developed UHPLC-MS-MS method (Yuan et al. 2013). Compound LF-3-99 (10 ng/mL, See Fig. S1 for structure) was used as an internal standard. Two selected reaction monitoring (SRM) transitions (quantifier and qualifier) were monitored for each compound with a dwell time of 50 msec/ion as follows: LF-3-88: *m/z* 276 to 70 and *m/z* 276 to 207; and LF-3-99: *m/z* 304 to 98.

#### Statistical analysis

Data were analyzed by one-way ANOVA followed by post hoc comparisons with Fisher's test when appropriate. Body weight data were analyzed using two-way repeated measures ANOVA, followed by Fisher's post hoc analysis. An effect was considered significant if *P* < 0.05. Statistical outliers that fell above or below 2 SD from the mean were removed from the final analysis.

## Results

### Radioligand-binding activities at CNS-related neurotransmitter receptors and transporters

The in vitro binding affinities of the compounds (LF-3-88, nicotine, and varenicline) are summarized in Table 1. LF-3-88 was found to have potent interactions with *β*2-containing nAChRs (*α*2*β*2, *α*3*β*2, *α*4*β*2, and *α*4*β*2*) with *K*_i_ values range of 0.6–15.4 nmol/L. It showed superior selectivity for *α*4*β*2- (*K*_i_ = 0.6 nmol/L) over *α*3*β*4- (*K*_i_ > 10^4^ nmol/L) and *α*7-nAChRs (*K*_i_ > 10^4^ nmol/L). In the broad-range screening study, no inhibition caused by 10 *μ*mol/L of compound LF-3-88 was greater than 50% across 51 other CNS neurotransmitter receptors and transporters, including serotonin receptors, dopamine receptors, GABA receptors, biogenic amine transporters, adrenergic receptors, muscarinic receptors, opioid receptors, sigma receptors, and histamine receptors, indicating that LF-3-88 has no significant activity at these targets. (see Table S1).

**Table 1:** Binding affinities at seven rat nAChR subtypes.

Compound	*K*_i_ (nmol/L)^1^								
	*α*2*β*2	*α*2*β*4	*α*3*β*2	*α*3*β*4	*α*4*β*2	*α*4*β*2^*^^2^	*α*4*β*4	*α*7	*α*7^*^^2^
LF-3-88^3^	1.0 ± 0.2	935	15.4 ± 4.1	>10^4^	0.6 ± 0.1	3.0 ± 0.4	1790	NA^4^	NA
Nicotine^5^	5.5	70.0	29.0	260	4.9	9.8	23.0	ND	ND
Varenicline^6^	–	–	–	86	0.4	–	110	125	–

nAChR, nicotinic acetylcholine receptor; ND: not detected.

1 See Materials and Methods.

2 *α*4*β*2^*^ or *α*7^*^, endogenous receptors prepared from rat forebrain. Besides *α*4, *β*2, or *α*7, other unidentified subunits may also be present. Details are provided in the Materials and Methods.

3 The *K*_i_ values for LF-3-88 are taken from a previous publication (Yu et al. 2012).

4 NA: not active, defined as < 50% binding in the primary assay at 10 *μ*mol/L.

5 The *K*_i_ values for nicotine are taken from the PDSP Assay Protocol Book.

6 The *K*_i_ values for varenicline are from the literature (Rollema et al. 2010).

### In vitro functional characterization

**Figure 2:**
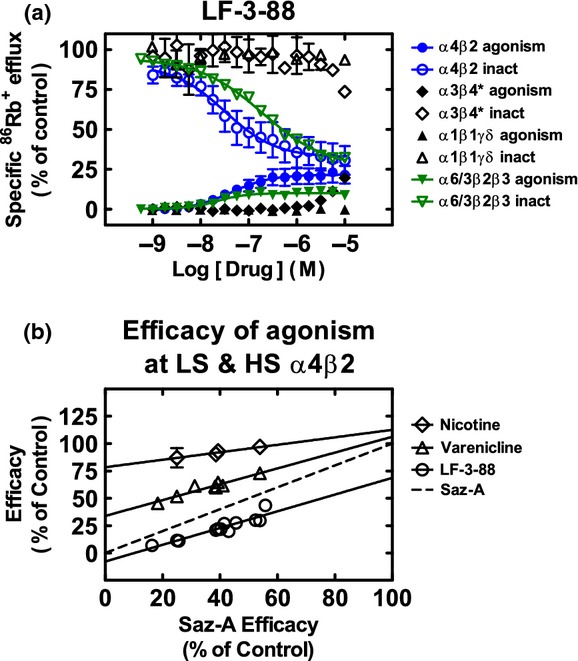
(A) Effects of LF-3-88 at nAChR subtypes. Specific ^86^Rb^+^ efflux (ordinate; percentage of control ± SEM) was determined as described in the Materials and Methods for intrinsic agonist activity over a 9.5 min period for compound LF-3-88 at the indicated concentrations (abscissa; log molar scale) on human *α*4*β*2-nAChR (●), (*α*6/3)*β*2*β*3-nAChR (▼), *α*3*β*4*-nAChR (♦), or *α*1*β*1*γδ*-nACHR (▲) naturally or heterologously expressed by SH-EP1-h*α*4*β*2, SH-EP1-h(*α*6/3)*β*2*β*3, SH-SY5Y, or TE671/RD cells, respectively. Also shown are functional inactivation effects of pretreatment for 10 min with the same agents at the indicated concentrations on subsequent agonist action of an EC_90_ concentration of the full agonist, carbamylcholine (applied in the continuing presence of the indicated agents), acting at *α*4*β*2-nAChR (○), (*α*6/3)*β*2*β*3-nAChR (▽), *α*3*β*4*-nAChR (♢), or *α*1*β*1*γδ*-nAChR (△). Results are normalized to responses to a fully efficacious concentration of carbamylcholine (see Materials and Methods for details). Nanomolar agonist EC_50_ values and inactivation IC_50_ values are provided in Table 2, as are agonism and inactivation efficacies (normalized to those for a full agonist or antagonist, respectively). (B) The unusual characteristic of sazetidine-A being a full agonist at HS *α*4*β*2-nAChRs while having zero or nearly zero efficacy at LS *α*4*β*2-nAChRs allows the determination of a ligand's efficacies at both HS- and LS-*α*4*β*2 in cells expressing a variable mix of the two *α*4*β*2-nAChR isoforms. The ordinate intercepts of the linear regressions at a sazetidine-A efficacy (abscissa) of 0% represent the efficacies at LS *α*4*β*2-nAChRs, while the ordinate intercepts at a sazetidine-A efficacy of 100% represent the efficacies at HS *α*4*β*2-nAChRs. Values and standard errors are reported in Table 2.

**Figure 3:**
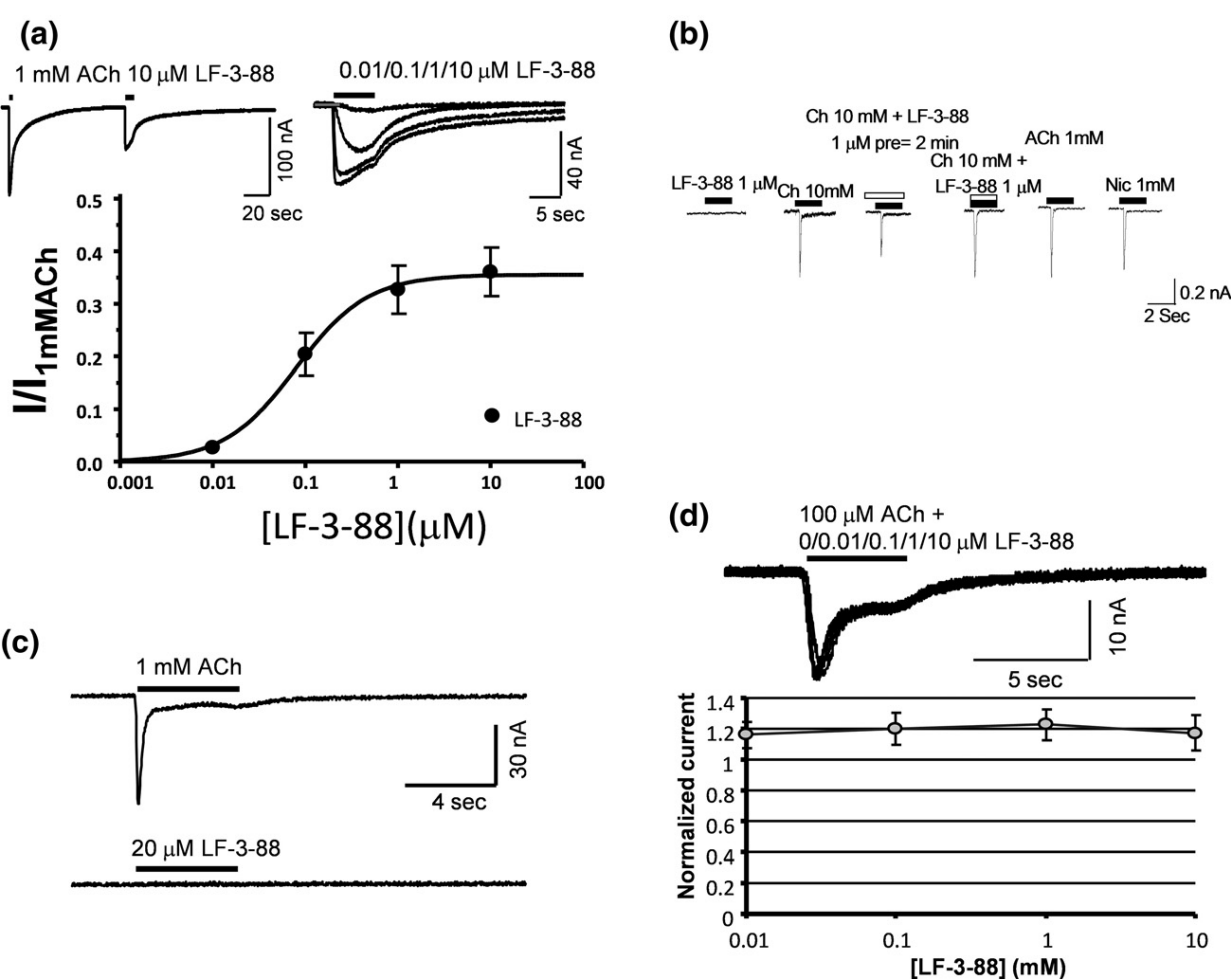
(A) Representative two-electrode voltage-clamp recording responses to 1 mmol/L acetylcholine (ACh) or to LF-3-88 at the indicated concentrations (for this and other panels, note time and current calibration bars), and dose–response relationship for LF-3-88 acting on human *α*4*β*2-nAChRs expressed in Xenopus oocytes. LF-3-88 is a partial agonist at *α*4*β*2-nAChRs. (B) Representative, sequential, whole-cell current recording responses of *α*7-nAChRs stably and heterologously expressed in the same SH-EP1-h*α*7 cells to (left-to-right) 1 *μ*mol/L LF-3-88; 10 mmol/L choline; 10 mmol/L choline during and after a 2 min pre-exposure to 1 *μ*mol/L LF-3-88; 10 mmol/L choline during coexposure to 1 *μ*mol/L LF-3-88; 10 mmol/L choline after the aforementioned drug exposures; 1 mmol/L nicotine after the aforementioned drug exposures, with 2 min intervals between drug applications. LF-3-88 has no agonist activity and only weakly at 1 *μ*mol/L inhibits functional *α*7-nAChRs. (C) Typical two-electrode voltage-clamp recording responses to 1 mmol/L acetylcholine (ACh) or to 20 *μ*mol/L LF-3-88 at the indicated concentrations of *α*7-nAChRs expressed in oocytes confirming lack of agonist activity. (D) Sample traces from two-electrode voltage-clamp recording of *α*7-nAChR responses to 100 *μ*mol/L ACh alone or in the presence of the indicated concentrations of LF-3-88 confirming weak if any antagonist activity of LF-3-88 on *α*7-nAChR expressed in Xenopus oocytes.

Functionally, LF-3-88 was characterized using three techniques: the ^86^Rb^+^ ion flux assay, two-electrode voltage-clamp (TEVC) recording in oocytes, and whole-cell current recording in SH-EP1-h*α*7 and SH-EP1-h*α*4*β*2 cells (Table 2; Figs. 2 and 3). Consistent with the binding assay results, LF-3-88 was selective for *α*4*β*2- and (*α*6/3)*β*2*β*3-nAChRs with EC_50_ values of 43 and 22 nmol/L, respectively, and IC_50_ values for inactivation of response to full agonists of 32 and 192 nmol/L, respectively, in the ^86^Rb^+^ ion flux assay (Table 2), while displaying no significant agonist or antagonist activity at ganglionic *α*3*β*4*- or muscle-type *α*1*β*1*γδ*-nAChRs. TEVC recordings in oocytes confirmed LF-3-88 agonism at *α*4*β*2-nAChRs with an EC_50_ of 79 nmol/L (Fig. 3A), and these findings were confirmed in whole-cell current recording from SH-EP1-h*α*4*β*2 cells (data not shown). At *α*7-nAChRs, whole-cell recordings revealed that with a 2-min pretreatment, LF-3-88 was only able to slightly inhibit activation by 10 mmol/L choline, but it showed no agonist activity and coapplication with choline had no effect (Fig. 3B). No effect at *α*7-nAChRs was detected in oocytes at concentrations as high as 20 *μ*mol/L (Fig. 3C and D). It is noteworthy that LF-3-88 exhibited potent partial agonism at HS *α*4*β*2 receptors with an efficacy that is 69% of that shown by carbamylcholine, but had little or no intrinsic activity at LS *α*4*β*2 receptors. Inactivation and/or desensitization of receptors by LF-3-88 to carbamylcholine stimulation was equipotent at HS and LS *α*4*β*2-nAChRs, as shown by the monophasic inhibition in Figure 2A. Nicotine and varenicline are provided for reference in Table 2. Noticeably, varenicline shows unusual properties for a high-affinity ligand with respect to agonism as measured with the ^86^Rb^+^ ion flux assay. This is due to the extended length of this endpoint assay which was performed to measure the clinically relevant IC_50_ for varenicline, which only reaches nanomolar concentrations in human brain at therapeutic doses (Weber et al. 2013).

**Table 2:** Potencies and efficacies of ligand agonism and inactivation of human *α*4*β*2-, *α*6/3*β*2*β*3-, and *α*3*β*4^*^-nAChRs^1^.

Compound		LF-3-88	Nicotine	Varenicline
Agonism *α*4*β*2-nAChR	EC_50_ (nmol/L)	43	295	938
	pEC_50_	7.37 ± 0.19	6.53 ± 0.05	6.03 ± 0.15
	HS Efficacy (%)	69 ± 5.6	124 ± 8.5	105 ± 4.4
	LS Efficacy (%)	−7.8 ± 3.8	70 ± 5.9	34 ± 2.8
Inactivation *α*4*β*2-nAChR	IC_50_ (nmol/L)	32	427	32
	pIC_50_	7.50 ± 0.26	6.37 ± 0.06	7.50 ± 0.26
	Efficacy (%)	68 ± 4.9	92 ± 2.1	79 ± 3.3
Agonism (*α*6/3)*β*2*β*3-nAChR	EC_50_ (nmol/L)	22.4	127	18
	pEC_50_	7.65 ± 0.06	6.9 ± 0.03	7.75 ± 0.07
	Efficacy	10 ± 0.27	56 ± 0.06	17 ± 0.5
Inactivation (*α*6/3)*β*2*β*3-nAChR	IC_50_ (nmol/L)	192	85	68
	pIC_50_	6.72 ± 0.07	7.07 ± 0.08	7.17 ± 0.08
	Efficacy (%)	74 ± 2.8	26 ± 1.4	74 ± 2.6
Agonism *α*3*β*4^*^-nAChR	EC_50_ (nmol/L)	ND	30000	2200
	Efficacy (%)	ND	90	110

nAChR, nicotinic acetylcholine receptor.

1 See Materials and Methods for details. The term “inactivation” is used because the compounds may be acting to desensitize receptors or as competitive or noncompetitive antagonists, and further work is needed to make such a distinction.

### Preliminary in vitro ADME-Tox profile

The metabolic stability of LF-3-88 was studied using human and mouse liver microsomes and cryopreserved hepatocytes (See Table S2). Incubation with liver microsomes for 1 h resulted in 81% (mouse) and 89% (human) of LF-3-88 remaining unchanged, while exposure to cryopreserved hepatocytes for 2 h resulted in 91% (mouse) and 92% (human) of LF-3-88 remaining unchanged. No significant inhibition was found toward five different cytochrome P450 (CYP450) enzymes (2D6, 3A, 1A, 2C9, and 2C19) as shown in the Table S3. Furthermore, LF-3-88 was found to be negative for mutagenic potential against tester strains TA98, TA100, and TA1535 with or without S9 cells present at four tested concentrations (5, 10, 50, or 100 *μ*mol/L) (See Table S4) in the Ames assays.

### Acute behavioral pharmacology

**Figure 4:**
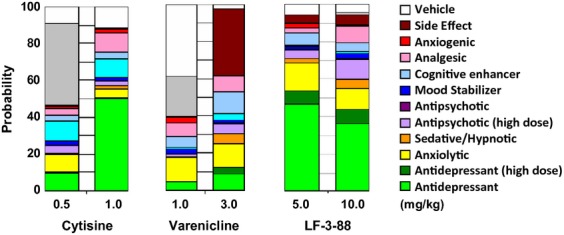
The behavioral SmartCube® signatures of cytisine, varenicline, and LF-3-88 are presented in different groups: antidepressants (green), anxiolytics (yellow), sedative (orange), antipsychotic (purple), mood stabilizer (blue), cognitive enhancer (light blue), analgesic (pink), anxiogenic (red), side effects (maroon), inactivation (similarity to vehicle, white), and signatures not recognized by the classifiers (gray).

The behavioral signatures in the SmartCube® assay of cytisine (0.5 and 1.0 mg/kg), varenicline (1.0 and 3.0 mg/kg), and LF-3-88 (5 and 10 mg/kg) after 15 min of preinjection (i.p.) are shown in Figure 4. All three of these nicotinic partial agonists were found to exhibit robust-mixed signatures at both of the tested doses. The large “green” component of the bar graphs were observed at 1.0 mg/kg for cytisine, and 5.0 and 10.0 mg/kg of LF-3-88, suggesting potential antidepressive-like behavior. Varenicline was found to show a side effect component with about 30% probability at 3.0 mg/kg as indicated by the maroon-colored component. Minimal side effects were found at 1.0 mg/kg of varenicline and all the tested doses of cytisine (0.5 and 1.0 mg/kg) and LF-3-88 (5 and 10 mg/kg).

**Figure 5:**
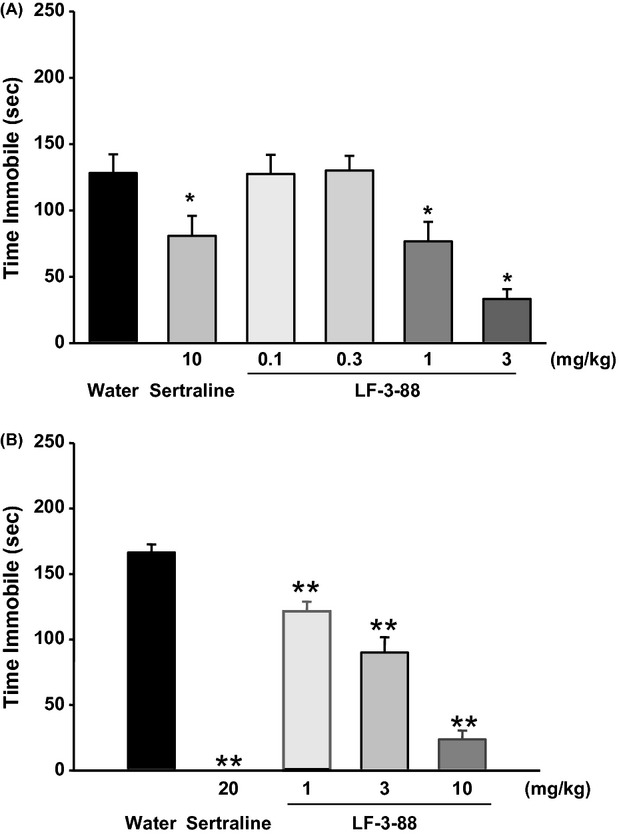
Effects of sertraline and LF-3-88 in mice on total time immobile in the FST (A, i.p.; B, p.o.). Data were summed over the 6-min test and represent mean ± SEM. *P < 0.05 and **P < 0.001 indicates statistical significance compared with water. ANOVAs: (A) *F*(5, 54) = 9.0, *P* < 0.001; (B) *F*(4, 43) = 78.3, *P* < 0.001. FST, forced swim test.

For forced swim tests, mice were administered compound LF-3-88 (0.1, 0.3, 1, and 3 mg/kg, i.p.) or the SSRI antidepressant, sertraline, as a positive control (10 mg/kg). Fisher's post hoc tests showed that compound LF-3-88 dose-dependently reduced immobility at 1 and 3 mg/kg compared with a water-treated control group, but not at 0.1 or 0.3 mg/kg, suggestive of a minimum effective dose of 1 mg/kg (Fig. 5A). Moreover, LF-3-88 was also found to reduce the immobility dose-dependently at an orally administered dose of 1, 3, and 10 mg/kg (Fig. 5B).

### Chronic novelty-suppressed feeding

**Figure 6:**
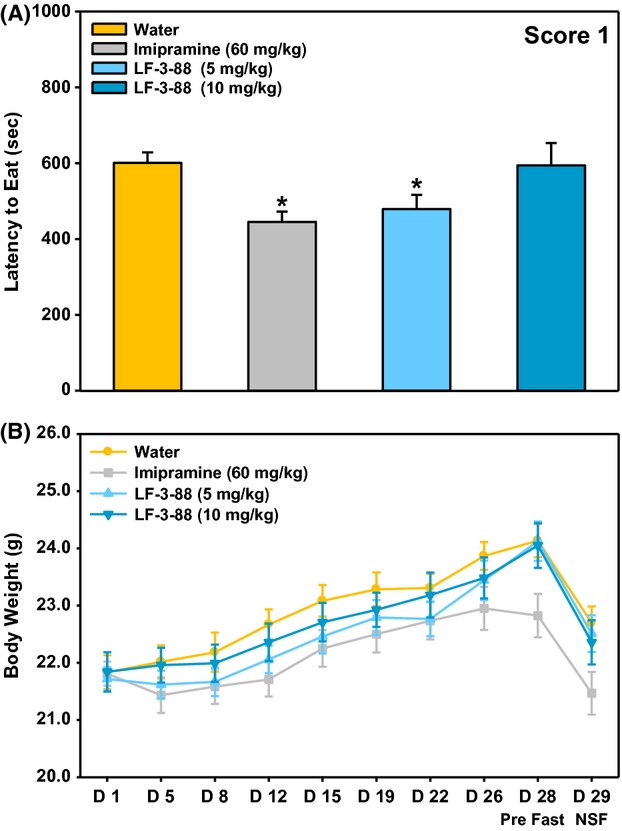
(A) Mean latency to eat during the 15-min test as scored by the first blinded observer. Data are presented as mean ± SEM; ANOVAs: *F*(3, 42) = 3.9, *P* < 0.05. (B) Average body weight across the 29 days of treatment. Data are presented as mean ± SEM; ANOVAs: *F*(3, 44) = 1.3. *Fisher's PLSD posthoc test: *P < 0.05 versus vehicle. *n* = 11 12/group.

Food-deprived mice experience a conflict between eating food placed in the center of an arena and being in an open, novel, bright space. This conflict results in a latency to eat that is reduced by chronic treatment with antidepressant compounds. This latency reduction is quantified and used in the experimental paradigm known as NSF. Chronic administration of the tricyclic antidepressant imipramine significantly decreased latency to eat compared with control group (Fig. 6A). LF-3-88 treatment at 5 mg/kg, but not at 10 mg/kg, significantly decreased latency to eat compared with results using water-treated control animals. No significant treatment effect on body weight was found for the 4-week administration of test compounds (repeated measures ANOVA; Fig. 6B). No obvious behavioral changes were observed in the NSF paradigm after 28 days oral administration of both 5 and 10 mg/kg doses, suggesting these doses were well-tolerated.

Mice were decapitated upon the completion of NSF, and trunk blood and whole-brain tissue were collected from all animals in the LF-3-88 treatment groups. Five blood samples and whole-brain tissues were chosen randomly for assessment. Plasma and brain levels of LF-3-88 were found to be 430 ± 172 ng/mL and 14 ± 4 ng/g at the treatment dose of 5 mg/kg and 942 ± 400 ng/mL and 31 ± 11 ng/g at the treatment dose of 10 mg/kg.

## Discussion

The cholinergic hypothesis of depression suggests that this debilitating mental disorder results from hyperactivity of the brain cholinergic system over the adrenergic system (Janowsky et al. 1972), suggesting pursuit of nAChR ligands as novel antidepressants. Recent Phase III findings that the nonselective nAChR antagonist TC-5214 (Targacept) failed to show efficacy as an adjunct therapy for patients that were nonresponders to traditional antidepressant treatment may indicate that nicotinic ligands are not good candidates for augmentation of SSRIs and SNRIs or that patients resistant to these therapies will also be resistant to drugs targeting nAChR, however, it seems most likely that TC-2514's principal inadequacy is insufficient potency (Weber et al. 2013). Coupled with poor selectivity, it would be unsurprising if a large enough dose to reach the desired pharmacological endpoint at *β*2*-nAChR in brain also caused undesirable side effects that would at best result in a narrow therapeutic window that is difficult to manage. It may also be that simple nAChR antagonism is not an optimal strategy to treat depressive symptoms in patients resistant to SSRIs and SNRIs. A number of potent and selective *α*4*β*2-nAChR partial agonists with distinct functional profiles have been reported to exhibit antidepressive-like behavior in vivo. In the present study, we characterized a selective *α*4*β*2- and *α*6*-nAChR partial agonist LF-3-88 with a favorable ADME-tox profile, and we evaluated its acute and chronic effects in a variety of behavioral paradigms. LF-3-88 is a potent *α*4*β*2-nAChR partial agonist with a *K*_i_ value similar to that of varenicline (0.6 nml/L vs. 0.4 nmol/L). However, LF-3-88 showed little or no binding affinity at *α*3*β*4- or *α*7-nAChRs (*K*_i_ > 10^4^ vs. 86 and 125 nmol/L of varenicline) or at 51 other CNS-related neurotransmitter receptors and transporters. Notably, LF-3-88 has relatively high efficacy at HS *α*4*β*2-nAChRs (69% relative to that of carbamylcholine), but little or no effect at LS receptors. This functional profile of LF-3-88 differs from that of other known *α*4*β*2-nAChR partial agonists such as cytisine and 3-(2-pyridinyl)cytisine that show low intrinsic efficacy of no more than 10% at both LS and HS receptors relative to that of ACh (Mineur et al. 2009).

Compound LF-3-88 showed a favorable profile in assays for CYP450 inhibition, metabolic stability, hERG (human ether-a-go-go-related gene) inhibition, and Ames toxicity. No significant inhibition was found against a panel of five different CYP450 enzymes (2D6, 3A, 1A, 2C9, and 2C19) suggesting a decreased risk for drug–drug interactions. Our previous pharmacokinetic study on LF-3-88 showed that the compound was absorbed rapidly and crossed the blood–brain barrier (BBB) in vivo (Yuan et al. 2013). Taken together, these preliminary ADME-Tox data encouraged us to conduct further studies in vivo.

In the SmartCube® test, cytisine, varenicline, and LF-3-88 exhibited robust CNS drug-like signatures. In particular, antidepressive-like behavior were most pronounced for LF-3-88 (5 and 10 mg/kg) and cytisine (1.0 mg/kg). In addition, the side effect signature of LF-3-88 was less than 10% at both tested doses, consistent with the good tolerability of these doses in the NSF. This contrasts with varenicline's profile, which showed a steep change from weak antidepressant effects at 1 mg/kg to significant side effects (about 30%) at 3.0 mg/kg. As it has been reported that cytisine is not tolerated by mice at a dose of >1.5 mg/kg (Mineur et al. 2009), we tested lower doses (0.5 and 1.0 mg/kg), and we observed no obvious side effects. The antidepressant activity of LF-3-88 was further confirmed using the classical mouse forced swim test, in which LF-3-88 was found to decrease the time spent immobile in a dose-dependent fashion with an estimated minimal effective dose of 1 mg/kg. We carried out a preliminary acute maximum tolerated dose study from which we found that LF-3-88 temporarily decreased temperature and active behaviors above 100 mg/kg but caused no serious toxic side effects or mortality up to 300 mg/kg in the C57/BL6J mice, in contrast with varenicline which is lethal at such doses when administered orally to Balb/C mice (data not shown). These preliminary mouse acute behavioral toxicity results indicate a reasonable therapeutic window.

In the NSF study, chronic administration of either the tricyclic antidepressant imipramine or LF-3-88 decreased latency to eat compared with the water-treated group. However, this effect of LF-3-88 was only observed at the lower dose (5 mg/kg) and was relatively weak. A similar phenomenon was previously observed in the case of 3-(2-pyridinyl)cytisine, where only administration of the lower dose of the compound (0.3 vs. 0.6 mg/kg) was found to be effective in the NSF test (Mineur et al. 2009). Many nicotinic ligands have been reported to decrease food intake and body weight, and as such may affect the tendency of mice to initiate feeding. In our study, we did not find a significant treatment effect on body weight for the 4-week administration of LF-3-88. A follow-up quantitative analysis of LF-3-88 in mouse plasma and brain tissue showed that LF-3-88 was in the brain and plasma and the exposure levels were dose dependent. One possible explanation could be that nicotinic ligands that bind to *α*4*β*2-nAChRs might have bimodal effects on anxiety. Nicotine has been found to be anxiolytic at lower doses while being anxiogenic at higher doses (File et al. 1998; Ouagazzal et al. 1999; Cheeta et al. 2001). Moreover, anxiolytic and anxiogenic effects of nicotine were observed at the same dose at different time points in the social interaction test (Irvine et al. 1999). In general the literature findings on the effects of nicotinic agonists in the chronic NSF paradigm have been inconclusive. In this test, nicotine failed to show significant behavioral changes after 28 days oral administration in both BALB/cJ and C57BL/6J mice (Vieyra-Reyes et al. 2008). After 14 days, repeat treatment in C57BL/6J mice, cytisine and 3-(2-pyridinyl)cytisine decreased the latency to eat; CP-601927 increased the latency to eat while varenicline did not show any effect (Mineur et al. 2009, 2011). Turner et al. (2010) found that chronic treatment with sazetidine-A decreased the time needed to approach food while no effect was observed in the case of varenicline with male 129SvJ;C57BL/6J F1 hybrid mice in the NIH (novelty-induced hypophagia) paradigm. Furthermore, differences in doses, routes of administration, pretreatment time, as well as pharmacokinetics properties of individual compounds may contribute to these contrasting data.

In conclusion, we have identified a selective *α*4*β*2- and *α*6*-nAChR partial agonist, LF-3-88, with distinct functional properties and a favorable ADME-tox profile. We have evaluated its antidepressive-like behavior in SmartCube, FST, and NSF mouse models. Although the antidepressant potential of LF is based on limited data, these data suggest that the brain-penetrable compound LF-3-88 is well-tolerated in both acute and chronic studies that it may be beneficial in balancing the agonism and antagonism of nAChRs that may be useful in clinical applications. These favorable properties of LF-3-88 were achieved while eliminating the acetylene moiety of the parent compound, Sazetidine-A, which is undesireable due to the theoretically possible conversion into a highly reactive oxirene.

## Abbreviations

ACh: acetylcholine
ADME-Tox: absorption, distribution, metabolism, excretion, and toxicity
BBB: blood–brain barrier
CNS: central nervous system
CYP: cytochrome P450
FST: forced swim test
hERG: human ether-a-go-go-related gene
HS: high sensitivity
LS: low sensitivity
nAChR(s): nicotinic acetylcholine receptor(s)
NIMH-PDSP: National Institute of Mental Health Psychoactive Drug Screening Program
NSF: novelty-suppressed feeding
SEM: standard error of the mean
SNRI: serotonin norepinephrine reuptake inhibitor
SRM: selected reaction monitoring
SSRI: selective serotonin reuptake inhibitor
TEVC: two-electrode voltage-clamp
TFA: trifluoroacetic acid

## Supporting Information

**Figure S1.** Structure of LF-3-99. **Table S1.** Primary binding competition efficacies (%) of compound LF-3-88 at 51 other neurotransmitter receptors and transporters^a^. **Table S2.** Metabolic stability of LF-3-88 at liver microsomes and hepatocytes. **Table S3.** Inhibition of LF-3-88 toward 5 CYP450 enzymes. **Table S4.** Genetic toxicity studies of LF-3-88. **Table S5.** Comparator standards.
