# Supplementary material for: The potent and selective α4β2*/α6*-nicotinic acetylcholine receptor partial agonist 2-[5-[5-((S)Azetidin-2-ylmethoxy)-3-pyridinyl]-3-isoxazolyl]ethanol demonstrates antidepressive-like behavior in animal models and a favorable ADME-tox profile
Source: Pharmacol Res Perspect. 2014 Mar 12;2(2):e00026. doi: 10.1002/prp2.26 (PMC4184702; doi:10.1002/prp2.26)
Supplement: Supplementary file 1 — Figure S1. Structure of LF-3-99. Table S1. Primary binding competition efficacies (%) of compound LF-3-88 at 51 other neurotransmitter receptors and transportersa. Table S2. Metabolic stability of LF-3-88 at liver microsomes and hepatocytes. Table S3. Inhibition of LF-3-88 toward 5 CYP450 enzymes. Table S4. Genetic toxicity studies of LF-3-88. Table S5. Comparator standards. [file prp20002-e00026-sd1.doc]

The Potent and Selective α4β2*/α6*-Nicotinic Acetylcholine Receptor (nAChR) Partial Agonist LF-3-88 Demonstrates Antidepressant-like Activity in Animal Models and a Favorable ADME-Tox profile

Li-Fang Yu,^†^ J. Brek Eaton,^‡^ Han-Kun Zhang, ^†^ Emily Sabath,^§^ Taleen Hanania, ^§^ Guan-Nan Li, ^†^ Richard B. van Breemen, ^†^ Paul Whiteaker, ^‡^ Qiang Liu,^#‡^ Jie Wu,^#^ Yong-Chang Chang, ^‡^ Ronald J. Lukas,^‡^ Dani Brunner^ǁ,§^ Alan P. Kozikowski ^*, †^

^†^ Department of Medicinal Chemistry and Pharmacognosy, University of Illinois at Chicago, 833 South Wood Street, Chicago, Illinois 60612, United States

^§^PsychoGenics, Inc., 765 Old Saw Mill River Road, Tarrytown, New York 10591, United States

^‡^Division of Neurobiology or ^#^Division of Neurology, Barrow Neurological Institute, 350 West Thomas Road, Phoenix, Arizona 85013, United States

^ǁ^Dept. of Psychiatry, Columbia University, NYSPI, 1051 Riverside Drive, New York 10032, United States

Phone: +1-312-996-7577; fax: +1-312-996-7107; e-mail: [kozikowa@uic.edu](mailto:kozikowa@uic.edu)

**List of Contents**

Broad Screening at Other Neurotransmitter Receptors and Transporters S2

Preliminary *in vitro* ADME-Tox S2

Structure of LF-3-99 S4

Comparator Standards S4

**Broad Screening at Other Neurotransmitter Receptors and Transporters**

**Table 1.** Primary binding competition efficacies (%) of compound **LF-3-88** at 51 other neurotransmitter receptors and transporters*^a^*

| Target | 5-HT_1A_ | 5-HT_1B_ | 5-HT_1D_ | 5-HT_1E_ | 5-HT_2A_ | 5-HT_2B_ | 5-HT_2C_ | 5-HT_3_ | 5-HT_4_ |
| --- | --- | --- | --- | --- | --- | --- | --- | --- | --- |
| Inhibition | 4.2 | -7.9 *^b^* | -14.9 | -3.0 | 34.1 | 7.9 | 22.7 | -1.0 | 3.7 |
| Target | 5-HT_5A_ | 5-HT_6_ | 5-HT_7_ | D_1_ | D_2_ | D_3_ | D_4_ | D_5_ | BZPR*^c^* |
| Inhibition | 13.2 | -1.9 | 1.1 | 5.8 | 17.2 | 3.0 | -2.5 | 5.8 | 6.7 |
| Target | α_1A_ | α_1B_ | α_1D_ | α_2A_ | α_2B_ | α_2C_ | β_1_ | β_2_ | β_3_ |
| Inhibition | -5.5 | -1.8 | -3.4 | 23.1 | -17.7 | 17.2 | 1.7 | 6.8 | 28.3 |
| Target | GABA_A_ | GABA_B_ | PBR | DAT*^e^* | NET*^e^* | SERT*^e^* | DOR*^d^* | KOR*^d^* | MOR*^d^* |
| Inhibition | -7.6 | 1.1 | -1.5 | 18.6 | 0.4 | -0.2 | 5.9 | 3.0 | 5.7 |
| Target | NMDA*^f^* | mGluR_5_ | M_1_ | M_2_ | M_3_ | M_4_ | M_5_ | H_1_ | H_2_ |
| Inhibition | -4.7 | 6.6 | 8.8 | 6.8 | 0.9 | -3.6 | -9.4 | -6.0 | 39.5 |
| Target | σ 1 | σ 2 | CB1 | CB2 | V1A | Oxytocin |  |  |  |
| Inhibition | 15.8 | 11.5 | 5.1 | -7.3 | 24.7 | 6.4 |  |  |  |

*^a^* The default concentration for primary binding experiments is 10 μM (n = 4). *^b^* Negative inhibition represents a stimulation of binding. *^c^* BZPR: Benzodiazepine Receptors (rat brain site). *^d^* DOR: Delta Opioid Receptor; KOR: Kappa Opioid Receptor; MOR: Mu Opioid Receptor. *^e^* DAT: Dopamine Transporter; NET: Norepinephrine Transporter; SERT: Serotonin Transporter. *^f^* NMDA: *N*-methyl-D-aspartate receptor.

**Preliminary *in vitro* ADME-Tox Profile**

**Table 2. Metabolic stability of LF-3-88 at liver microsomes and hepatocytes.**

| Metabolic stability Assay | Concentration (M) | Remaining |
| --- | --- | --- |
| liver microsomes, mouse, CD-1 | 1.0E-06 | 81 % |
| liver microsomes, human | 1.0E-06 | 89 % |
| cryopreserved hepatocytes, human | 1.0E-06 | 92 % |
| cryopreserved hepatocytes, mouse, CD-1 | 1.0E-06 | 91 % |

**Table 3. Inhibition of LF-3-88 toward 5 CYP450 enzymes.**

| CYP Assay | Concentration (M) | Inhibition (%) |
| --- | --- | --- |
| CYP2D6 inhibition, HLM, dextromethorphan substrate | 1.0E-05 | -0.4 |
| CYP3A inhibition, HLM, midazolam substrate | 1.0E-05 | 1 |
| CYP1A inhibition, HLM, phenacetin substrate | 1.0E-05 | 13 |
| CYP2C9 inhibition, HLM, diclofenac substrate | 1.0E-05 | 4 |
| CYP2C19 inhibition, HLM, omeprazole substrate | 1.0E-05 | 10 |

**Table 4.** Genetic toxicity studies of LF-3-88.

| Genetic Toxicity Assay | Concentration (M) | Cytotoxicity (% of Control Values) |
| --- | --- | --- |
| Bacterial cytotoxicity  (strain TA98 – S9) | 6.25E-07 | 97 |
|  | 1.25E-06 | 95 |
|  | 2.5E-06 | 98 |
|  | 5.0E-06 | 96 |
|  | 1.0E-05 | 92 |
|  | 2.5E-05 | 94 |
|  | 5.0E-05 | 102 |
|  | 1.0E-04 | 108 |
| Bacterial cytotoxicity  (strain TA100 – S9) | 6.25E-07 | 101 |
|  | 1.25E-06 | 102 |
|  | 2.5E-06 | 99 |
|  | 5.0E-06 | 103 |
|  | 1.0E-05 | 103 |
|  | 2.5E-05 | 115 |
|  | 5.0E-05 | 125 |
|  | 1.0E-04 | 139 |
| Bacterial cytotoxicity  (strain TA1535 – S9) | 6.25E-07 | 99 |
|  | 1.25E-06 | 105 |
|  | 2.5E-06 | 102 |
|  | 5.0E-06 | 100 |
|  | 1.0E-05 | 97 |
|  | 2.5E-05 | 104 |
|  | 5.0E-05 | 109 |
|  | 1.0E-04 | 117 |
| Genetic Toxicity Assay | Concentration  (M) | Result |
| Ames test  (strain TA98 – S9) | 5.0E-06 | Negative |
|  | 1.0E-05 | Negative |
|  | 5.0E-05 | Negative |
|  | 1.0E-04 | Negative |
| Ames test  (strain TA98 + S9) | 5.0E-06 | Negative |
|  | 1.0E-05 | Negative |
|  | 5.0E-05 | Negative |
|  | 1.0E-04 | Negative |
| Ames test  (strain TA100 – S9) | 5.0E-06 | Negative |
|  | 1.0E-05 | Negative |
|  | 5.0E-05 | Negative |
|  | 1.0E-04 | Negative |
| Ames test  (strain TA100 + S9) | 5.0E-06 | Negative |
|  | 1.0E-05 | Negative |
|  | 5.0E-05 | Negative |
|  | 1.0E-04 | Negative |
| Ames test  (strain TA1535 – S9) | 5.0E-06 | Negative |
|  | 1.0E-05 | Negative |
|  | 5.0E-05 | Negative |
|  | 1.0E-04 | Negative |
| Ames test  (strain TA1535 + S9) | 5.0E-06 | Negative |
|  | 1.0E-05 | Negative |
|  | 5.0E-05 | Negative |
|  | 1.0E-04 | Negative |

Fig. 1. Structure of LF-3-99.

**Table 5.** Comparator Standards.

| Assays | Comparator Standards |
| --- | --- |
| Radioligand binding assays | See PDSP protocol book http://pdsp.med.unc.edu/ |
| ^86^Rb ion efflux assays | Sazetidine-A, nicotine, varenicline |
| Whole-cell current recording assays | Choline, nicotine |
| Two-electrode voltage-clamp | Acetylcholine |
| SmartCube | Cytisine and varenicline |
| Forced swim tests | Sertraline |
| Novelty-suppressed feeding tests | Imipramine |
| Metabolic stability  (liver microsomes, human) | Imipramine, propranolol, terfenadine, verapamil |
| Metabolic stability  (human and mouse, CD1) | Imipramine, propranolol, terfenadine, verapamil |
| Metabolic stability  (cryopreserved hepatocytes, human) | Flurazepam, HFC, naloxone, propranolol |
| Metabolic stability  (cryopreserved hepatocytes, mouse, CD1) | Flurazepam, HFC, naloxone, propranolol |
| CYP1A inhibition (HLM, phenacetin sybstrate) | Furafylline |
| CYP2C9 inhibition (HLM, diclofenac substrate) | Sulfaphenazole |
| CYP2C19 inhibition (HLM, omeprazole sybstrate) | Oxybutynin |
| CYP2D6 inhibition (HLM, dextromethorphan substrate) | Quinidine |
| CYP3A inhibition (HLM, midazolam substrate) | Ketoconazole |
| CYP3A inhibition (HLM, testosterone substrate) | Ketoconazole |
| Bacterial cytotoxicity assays | Mitomycin C |
| Ames tests | Aminoanthracene, mitomycin C, quercetin, streptozotocin |
